# Effectiveness of Home-Based Telerehabilitation Interventions for Dysphagia in Patients With Head and Neck Cancer: Systematic Review

**DOI:** 10.2196/47324

**Published:** 2023-09-08

**Authors:** Wenwen Yang, Yifei Du, Mengran Chen, Sufang Li, Fan Zhang, Peiyang Yu, Xiaoxia Xu

**Affiliations:** 1 The Affiliated Cancer Hospital of Zhengzhou University & Henan Cancer Hospital Zhengzhou City, Henan Province China

**Keywords:** head and neck cancer, home-based rehabilitation, remote intervention, swallowing exercise, systematic review

## Abstract

**Background:**

Multimodal treatment–induced dysphagia has serious negative effects on survivors of head and neck cancer. Owing to advances in communication technologies, several studies have applied telecommunication-based interventions that incorporate swallowing exercises, education, monitoring, feedback, self-management, and communication. It is especially urgent to implement home-based remote rehabilitation in the context of the COVID-19 pandemic. However, the optimal strategy and effectiveness of remote interventions are unclear.

**Objective:**

This systematic review aimed to examine the evidence regarding the efficacy of telerehabilitation for reducing physiological and functional impairments related to swallowing and for improving adherence and related influencing factors among head and neck cancer survivors.

**Methods:**

The PubMed, MEDLINE, CINAHL, Embase, and Cochrane Library databases were systematically searched up to July 2023 to identify relevant articles. In total, 2 investigators independently extracted the data and assessed the methodological quality of the included studies using the quality assessment tool of the Joanna Briggs Institute.

**Results:**

A total of 1465 articles were initially identified; ultimately, 13 (0.89%) were included in the systematic review. The quality assessment indicated that the included studies were of moderate to good quality. The results showed that home-based telerehabilitation improved the safety of swallowing and oral feeding, nutritional status, and swallowing-related quality of life; reduced negative emotions; improved swallowing rehabilitation adherence; was rated by participants as highly satisfactory and supportive; and was cost-effective. In addition, this review investigated factors that influenced the efficacy of telerehabilitation, which included striking a balance among swallowing training strategy, intensity, frequency, duration, and individual motor ability; treating side effects of radiotherapy; providing access to medical, motivational, and educational information; providing feedback on training; providing communication and support from speech pathologists, families, and other survivors; and addressing technical problems.

**Conclusions:**

Home-based telerehabilitation has shown great potential in reducing the safety risks of swallowing and oral feeding, improving quality of life and adherence, and meeting information needs for dysphagia among survivors of head and neck cancer. However, this review highlights limitations in the current literature, and the current research is in its infancy. In addition, owing to the diversity of patient sociodemographic, medical, physiological and functional swallowing, and behavioral factors, we recommend the development of tailored telemedicine interventions to achieve the best rehabilitation effects with the fewest and most precise interventions.

## Introduction

### Background

Head and neck cancer (HNC) was the seventh most common cancer in the world in 2020, with 932,000 new cases and 468,000 related deaths [1]. Two-thirds of patients with HNC are at an advanced stage at the time of confirmed diagnosis, and these individuals often experience severely impaired physiological functioning. The 5-year survival rate among patients with HNC who undergo multimodal treatment (chemotherapy, radiation therapy, and surgery) is still <40% [2-4].

Moreover, the radiation-induced cascade of fibrosis and muscle atrophy in the swallowing muscle tissue [5,6] can cause severe damage to the nerves and organs associated with swallowing. The prevalence of dysphagia during and after treatment reaches 60% to 95%. In addition, the impaired swallowing barrier and decreased neuromuscular sensitivity result in aspiration, cough, and other complications [7]. Bacteria mistakenly inhaled into the nasopharynx or oropharynx can cause aspiration pneumonia upon entering the lung, which seriously threatens the safety of survivors [8]. When dysphagia is severe, patients avoid oral feeding and rely on gavage to reduce the risk of aspiration. However, total dependence on tube feeding leads to oropharyngeal disuse atrophy and further degeneration of the swallowing muscle tissue, resulting in malnutrition and low immunity and further leading to a risk of infection, falls, or other injuries [9]. The quality of life of HNC survivors has been significantly reduced owing to the severe effects of dysphagia [10], and anxiety and depression in patients with HNC are also higher than in patients with other cancers [11,12].

In recent years, owing to advances in swallowing therapy, rehabilitation exercises, and management strategies for HNC survivors, various intervention strategies have been developed to provide patients with multiple options for swallowing intervention. These interventions have been developed in close cooperation with multidisciplinary health care professionals and led by speech pathologists. However, the COVID-19 pandemic has created an urgent need for high-quality remote rehabilitation as patient safety and virus mitigation have made home-based telemedicine a priority for health care systems worldwide [13]. The future of rehabilitation medicine lies in *comfort medicine*, and patients’ sense of gain comes from comfort [14,15]. Therefore, speech therapists need to build a structured family rehabilitation practice model to strengthen the prevention and treatment of dysphagia with efficient, convenient, and comfortable rehabilitation management. To ensure swallowing safety, oral feeding should be achieved as soon as possible, and the risk of malnutrition caused by dependence on gavage should be reduced, thereby improving the quality of life and reducing the prevalence of anxiety, depression, and other negative emotions [10,16-18].

However, given the variability in intervention designs and the diversity of web resources and support strategies for the telerehabilitation of HNC survivors, the benefits and barriers of telerehabilitation strategies remain unknown. Determining the benefits of remote rehabilitation requires not only confirming improvements in swallowing physiology and function but also the satisfaction of patients’ health information needs. The satisfaction of health information needs can relieve patients’ anxiety, fear, depression, and other emotions; improve the degree of disease knowledge; reduce disease uncertainty; increase the satisfaction of participating in treatment decision-making; and improve the treatment effect and long-term health-related quality of life. In addition, given that low adherence will exert a significant effect on the outcomes of telerehabilitation, the factors that promote and hinder patient adherence must be assessed.

### Aims and Objectives

The aims of this review were 2-fold. First, we aimed to determine whether remote rehabilitation interventions for HNC survivors produce the expected benefits; second, we aimed to summarize the factors that influence the efficacy of telerehabilitation. Clarifying the effectiveness and factors influencing the home-based telerehabilitation model provides an evidence-based framework for the treatment and management of dysphagia in patients with HNC by speech-language pathologists and nurses and the generalizability of these results to other telemedicine services.

## Methods

### Data Sources and Search Strategy

We systematically searched the PubMed, MEDLINE, CINAHL, Embase, and Cochrane Library databases up to July 2023. The search terms included “HNC,” “remote rehabilitation,” “telemedicine,” and “dysphagia.” The search terms for each database are listed in Multimedia Appendix 1 [10,18-29]. The reference lists of relevant articles and systematic reviews were also manually searched to identify potentially eligible studies.

### Study Selection

The retrieved articles were independently screened and evaluated by WY and YD. The titles and abstracts were screened in accordance with the inclusion criteria. Studies that examined different populations, applied different interventions, or evaluated different outcomes were excluded. Moreover, duplicate reports, reviews, guidelines, opinion papers, and summaries of meetings were excluded. After identifying potentially relevant studies, the full texts were obtained and reviewed. Studies with unavailable, insufficient, or unreported information on a remote intervention were excluded. In addition, studies that did not include data on the effects of swallowing rehabilitation and its supporting factors were excluded. Any discrepancies between the 2 reviewers were resolved through discussion with MC.

### Inclusion Criteria

The systematic review included studies that met the following 4 criteria.

#### Participants

The study population was patients diagnosed with HNC (originating from the mouth, oropharynx [soft palate, palatine tonsil, and lingual tonsil or tongue root], nasopharynx, nasal cavity or sinus, salivary glands, hypopharynx, and larynx). There were no restrictions regarding cancer location or stage. We excluded patients with cognitive deficits; dysphagia of mixed etiology; severe complications (such as pharyngeal fistula and infection); or severe cardiac, liver, lung, or other cachexia.

#### Intervention

Interventions could be any home-based swallowing intervention (including some home exercise programs) provided remotely through information and communications technology. Relevant studies included research on evaluation, prevention, intervention, monitoring, education, and counseling provided via telephone, internet, applications, virtual reality, storage and forwarding of communications, and real-time interaction with data collected via sensors or wearable devices.

#### Comparator

The control groups included individuals in routine nursing care, basic training, or other types of interventions (not limited).

#### Outcomes

The outcomes included the impact of home-based telerehabilitation on swallowing physiology and function, nutritional status, quality of life, adherence to treatment plans, and cost-effectiveness in patients with HNC or factors associated with the implementation of telerehabilitation.

### Data Extraction

Textbox 1 shows the data that were extracted from each article by 2 independent researchers using a predesigned extraction table. The data were extracted in accordance with the PRISMA (Preferred Reporting Items for Systematic Reviews and Meta-Analyses) guidelines.

If the published data were insufficient, the appropriate authors were contacted and asked to provide the missing information. Differences were resolved through discussion or, when necessary, consultation with a third reviewer.

**Data extraction.**

**Study details**

- Title, first author, year, and study type

**Participant inclusion and exclusion criteria**

- Participant details: age, sex, tumor location, tumor stage, and prescribed treatment
- Enrollment details: number of individuals screened, qualified, randomized, and finalized interventions

**Methodological quality**

- A quality assessment tool from the Joanna Briggs Institute (2016)

**Intervention details**

- Telerehabilitation or telemedicine technology, equipment, or tools
- Persons involved in the intervention, intervention timing (before, during, or after treatment), intervention duration, and follow-up interval
- Type of rehabilitation exercise, exercise duration, exercise frequency, exercise intensity, and comparison of interventions

**Measurement of results**

- Measurement tools used, by whom, when, and how (in person or through information and communications technology)

**Main results**

- Physiology and functioning of swallowing (swallowing safety, swallowing muscle activity, and oral feeding safety), nutritional status, swallowing-related quality of life, swallowing-related negative emotions, satisfaction with remote rehabilitation, adherence to remote rehabilitation, and barriers and benefits of remote rehabilitation

### Quality Assessment

In total, 2 reviewers used the quality assessment tool of the Joanna Briggs Institute (2016) to independently assess the methodological quality of each study, as shown in Multimedia Appendix 1 [10,18-29]. For each evaluated item, the 2 researchers made judgments of “Yes,” “No,” “Not clear,” or “Not applicable.” In addition, overall methodological quality was assigned to each study based on the sum of the evaluated items (“No”=0, “Yes”=1, and “Not clear” and “Not applicable”=not scored). The methodological quality of each study ranged from 0 (lowest) to 13 (highest) points. Group discussions were held to determine whether additional information was necessary for each study.

## Results

### Search Process

**Figure 1:**
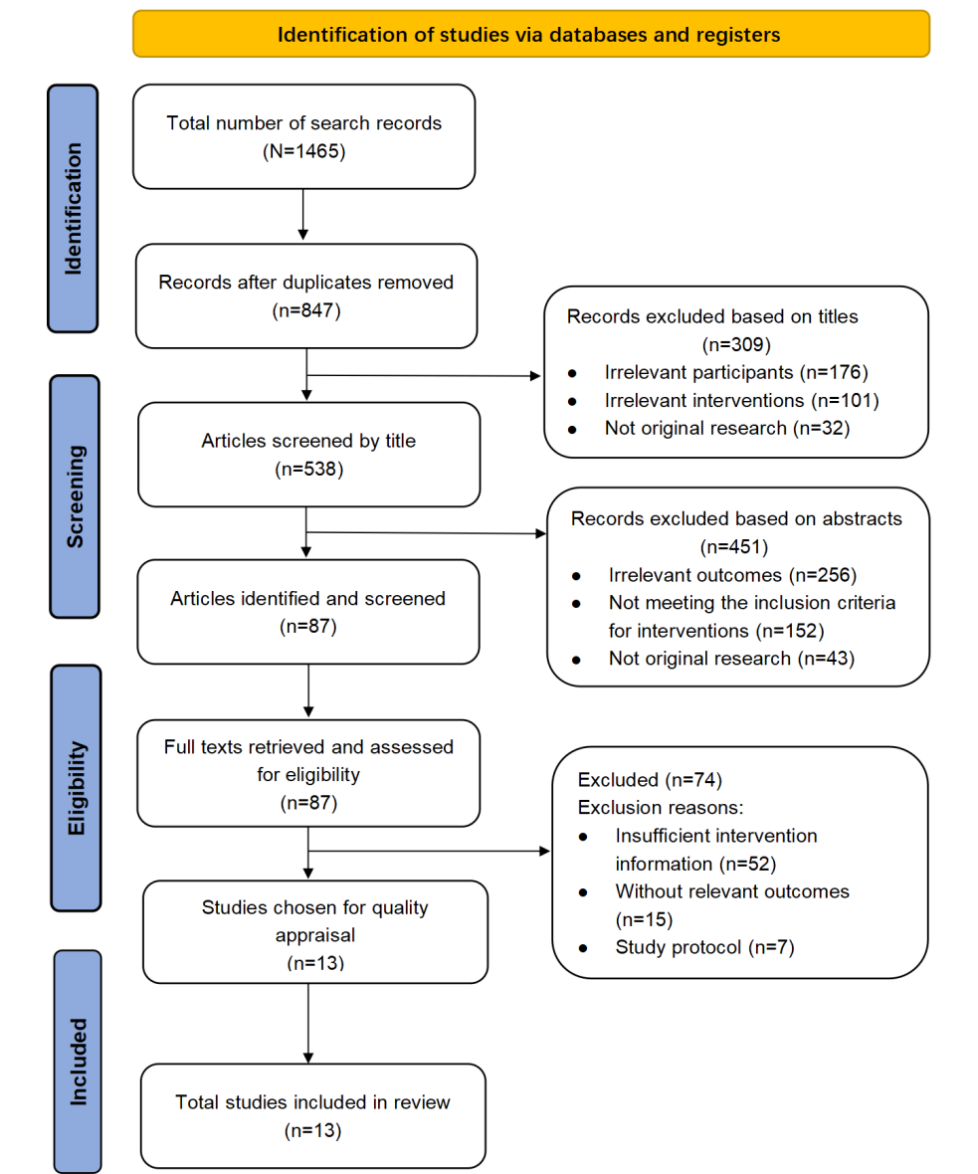
Selection process for the original studies.

The search and selection process for the included studies is shown in Figure 1. We identified 1465 studies; reviewed the full texts of 87 (5.94%); and, ultimately, included 13 (0.89%) in this systematic review.

### Methodological Quality Assessment

Detailed results of the methodological quality assessment are presented for each included study in Multimedia Appendix 1 [10,18-29]. All randomized controlled trials (RCTs) had an unclear risk of bias in terms of the blinding of participants or providers. Except for 15% (2/13) of the studies [19,20], no investigator reported that they used blinding methods when obtaining the results. A total of 31% (4/13) of experimental studies lacked a control group. Regarding the treatment of missing data, the studies indicated that intention-to-treat or sensitivity analyses were used. As all studies scored between 5 and 11 points on the risk-of-bias assessment, they were considered to be of intermediate quality. Therefore, no studies were excluded based on quality assessment scores. However, the type, content, focus, and outcome measurement indicators of remote interventions varied among the studies, resulting in heterogeneity.

### Study Characteristics

#### Overview

In this systematic review, among the 13 studies, we included 7 (54%) RCTs, 5 (38%) quasi-experimental studies, and 1 (8%) cohort study, encompassing a total of 1208 participants and 914 participants who completed their trials.

The studies were conducted in the Netherlands, Denmark, Belgium, the United States, Australia, China, and Canada. All the studies (13/13, 100%) were published in English between 2014 and 2023. The sample sizes of the studies ranged from 20 to 235, and the sample size of 54% (7/13) of the studies was <100. The mean age of the participants was 57 to 65 years. Most of the participants were male, and the sex ratio was approximately 2.23:1. The tumor stages were mainly III and IV. The most common tumor site was the oropharynx, followed by the larynx. See Table S1 in Multimedia Appendix 2 [10,18-29] for details.

#### Intervention Information

Table S2 in Multimedia Appendix 2 [10,18-29] summarizes the characteristics of the interventions used in the included studies.

#### Intervention Types

The types of information and communications technologies used to provide telerehabilitation interventions varied among the studies. Of the 13 studies included, 11 (85%) used asynchronous health care primarily and supplemented by synchronous health care, and 2 (15%) reported a comparison between asynchronous and synchronous telemedicine.

#### Asynchronous Management Supports

Most studies (8/13, 62%) used an application-based asynchronous remote intervention. For example, in a study that used a WeChat-based intervention, in addition to providing education and information on rehabilitation training, the members of the intervention team regularly communicated with the patients. This 2-way and internet-based communication allowed patients to accurately express their training status to better adjust exercise intensity according to the rehabilitation situation [20]. A total of 15% (2/13) of the studies used the *In Tune without Cords* application for customized rehabilitation regarding swallowing and lymphedema in patients with HNC and provided self-care education [21,22]. In total, 15% (2/13) of the studies used the *Vibrent and Head and Neck Virtual Coach* mobile app to provide personalized swallowing training and interactive queries to improve the adherence of patients to swallowing rehabilitation [10,18]. Another study used techniques such as wearable sensor–based biofeedback systems and the application of *Mobili-T* for remote swallowing rehabilitation interventions and remote monitoring (surface electromyography signals collected by sensor devices) [23].

A second method involved website-based asynchronous rehabilitation interventions. A total of 15% (2/13) of the studies provided tailored interactive feedback for participants in a web multimodal guided self-help exercise program (*Head Matters*) over the internet [24,25]. One study used a network-based platform (*PREPARE*) to provide swallowing and psychosocial intervention through personalized self-management via coping strategies according to the chemoradiotherapy cycle [26].

#### Synchronous Management Supports

In all the included studies (13/13, 100%), participants were instructed, educated, encouraged, supported, and supervised by telephone as they underwent remote rehabilitation training. Another study provided participants with home-based telemedicine via video calls, including assessment and rehabilitation of swallowing and speech functions, nutrition management, and investigation of side effects after HNC treatment [27].

#### Asynchronous Versus Synchronous Management Supports

An RCT compared 3 modes of service delivery: face-to-face therapy, the interactive *SwallowIT* application, and patient self-directed therapy. Compared with the clinician-guided model, swallowing effect, nutrition, and adherence were higher in the *SwallowIT* application than in the patient-guided group [29]. Another RCT also compared 3 modes of service delivery for preventive swallowing training, including diary support, app support, and therapist support. Their results were reversed, with lower adherence rates in the app and paper groups than in the therapist group, and adherence in the app group was lower than that in the paper group at weeks 3 and 4 [28].

#### Intervention Duration, Intensity, and Frequency

All the included studies (13/13, 100%) administered interventions with durations of 6 to 12 weeks, and 8% (1/13) of the studies administered an intervention for up to 6 months [20]. Of the 13 included studies, 8 (62%) recommended daily training, 5 (38%) recommended 15-minute–intensity training 3 times a day [19,21,22,24,25], 2 (15%) involved twice daily training [10,26], and 1 (8%) involved once daily training [23].

#### Intervention Modules

All the included studies (13/13, 100%) involved home-based remote rehabilitation. The specific intervention modules are shown in Table 1 and were divided into 7 categories: pretreatment guidance, swallowing rehabilitation training, educational information, experience sharing, training records, training reminders, remote monitoring, and long-term follow-up.

**Table 1:** Content of various intervention modules.

Module	Format	Description and function
Pretreatment instruction	Face-to-face educational meeting Video or telephone call Written explanation	Expert presentations: provided information on HNC^a^ therapy (surgical treatment, chemotherapy, radiotherapy, and rehabilitation exercises), informed patients of possible swallowing problems and side effects of chemoradiotherapy during treatment, and encouraged patients to adhere to rehabilitation training Presentations of swallowing practices by swallowing therapists each rehabilitation session: provided participants with an adequate description of each session and provided an instruction manual containing general information about HNC, rehabilitation photos, and a video presentation Educational content: helped participants log into a website account or helped them download, install, and use apps on selected mobile devices (cell phones and tablets) and reviewed the use and functionality of the site or app until participants demonstrated proficiency
Swallowing rehabilitation	Websites App Video or telephone call Photo, text, and video examples	Lip training program: resistance training Mandibular training program: mouth opening training, mandibular resistance training, and mandibular range of motion Cheek training program: lip lick, lip shrink, pouting, and eating from the affected side to the healthy side Tongue training program: swallowing hard, intensive tongue training, tongue resistance training, tongue pressing and maintenance of tongue motion, and Masako training and tongue retraction (tongue retraction, simulated gargle, and simulated yawn) Throat training program: Mendelsohn exercise, shaking exercise, mandibular resistance training, hard pitch sliding, supraglottic swallowing, and inspiratory and expiratory muscle strength training Neck and shoulder training: moving shoulders up and down and rotating the neck and shoulder back and forth
Information education	Websites App Video or telephone call Written explanation	Rehabilitation information: swallowing rehabilitation training video, audio and picture links were tailored to the treatment cycle of the patient, and high-quality educational website links and research literature that support patients at specific treatment points were used at the patient’s discretion Medical information: provision of information about HNC, HNC treatment, side effects of radiotherapy, pain information, airway management, speech, smell, nutrition, self-care advice, hospice care, and any other support personnel or cancer treatment facility information Oral care: a video presentation by the surgeon, self-assessment of dry mouth symptoms, tips for managing dry mouth, and food advice for dry mouth
Experience sharing	Websites App	Personal stories of patients who experienced swallowing function recovery were provided to improve adherence Participants joined the rehabilitation team through the app or website and could share information on their posttreatment life and treatment experience
Training records	Websites App	Self-recording: patients recorded daily which recovery exercises were performed, how often they were performed, and how hard each exercise felt when it was completed, and they recorded their treatment information and symptoms, including the date, start time of radiation therapy, body temperature, body weight, extent of pain, diet (type of food and amount ingested), oral ulcers, vomiting, skin reactions, and diarrhea
Training reminder	Phone or email Websites App	Speech pathologists: during rehabilitation interventions, participants attended a 10-minute weekly SLP^b^ coaching session by phone or email to maintain their motivation and improve treatment adherence Sites or apps: provided reminders to start and complete training each day and to return to hospital for treatment and follow-up
Remote monitoring	Sensor biofeedback Websites App	All data using web-or app-based records were provided web to enable health care providers to provide tailored internet-based feedback to each patient; if the training was not completed, an interactive query was sent to determine the reason for nonadherence; communication between the patient and the provider could occur via a messaging system sEMG^c^ signals collected by the sensor device were transmitted to the app via Bluetooth, and training was monitored remotely (the system defined table EMG^d^ data as representing a single swallow)
Long-term follow-up	Video or telephone call Face-to-face consultation Websites App	A video presentation of the oncologist self-examination with links to suggested survival care plans for follow-up Participants were evaluated for current smoking and alcohol use

^a^HNC: head and neck cancer.

^b^SLP: speech-language pathologist.

^c^sEMG: surface electromyography.

^d^EMG: electromyography.

### Assessment Tools

#### Swallowing Function

A total of 15% (2/13) of the studies assessed the safety of swallowing (aspiration, penetration, and pharyngeal residue) in patients with HNC using subjective and objective methods involving instruments and scales. Hajdú et al [19] assessed swallowing function using fiberoptic endoscopic evaluation of swallowing (FEES) in combination with the penetration-aspiration scale (PAS) and the Yale Pharyngeal Residue Severity Rating Scale. Wall et al [29] assessed swallowing function using the modified barium swallow study (MBS) and videofluoroscopic swallow study (VFSS) in combination with the PAS. A total of 23% (3/13) of the studies evaluated the maximum mouth opening distance in patients [19,25,29]. In total, 8% (1/13) of the studies evaluated the range of motion of swallowing muscles using myoelectric signals [23].

#### Dietary and Nutritional Status

In total, 15% (2/13) of the studies used the Functional Oral Intake Scale to assess oral feeding [19,29], and 8% (1/13) of the studies used the Patient-Generated Subjective Global Assessment to assess nutritional status [29].

#### Quality of Life

A total of 7 different assessment tools were used in the included studies: the MD Anderson Dysphagia Inventory [19,23,26,29], the European Organisation for Research and Treatment of Cancer (EORTC) Core Quality of Life Questionnaire [19,21,22,30], the EORTC Quality of Life Questionnaire–Head and Neck 35 [19,21,22,25,30,31], the Functional Assessment of Cancer Therapy–Head and Neck [29], the Swallowing Quality of Life questionnaire [21,22], the EQ-5D-3L questionnaire [21,22], the EORTC QLU-C10D [21,22], and the Assessment of Quality of Life-6-domain [29].

#### Patients’ Negative Emotions

Hajdú et al [19] used the Major Depression Inventory and 92-item Symptom Checklist anxiety subscale to assess negative emotions. Wall et al [29] used the Hospital Anxiety and Depression Scale to assess negative emotions.

#### Adherence

In total, 85% (11/13) of the studies evaluated adherence to home-based swallowing telerehabilitation and investigated facilitators of and barriers to rehabilitation training [10,18,19,21-26,28,29].

#### Patient Satisfaction

A total of 15% (2/13) of the studies evaluated patient satisfaction using the Functional Assessment of Chronic Illness Therapy–General Treatment Satisfaction [27,29,32].

#### Cost-Benefit

A cost-benefit analysis of remote swallowing rehabilitation interventions was conducted in 8% (1/13) of the studies. Jansen et al [21,22] evaluated the cost-benefit trade-off using the Institute for Medical Technology Assessment Medical Consumption Questionnaire and Productivity Cost Questionnaire.

### Telerehabilitation Effect

This section describes the intervention effects of home-based swallowing telerehabilitation in terms of the following 7 categories: physiology and function of swallowing, nutritional status, swallowing-related quality of life, swallowing-related negative emotions, adherence to exercise, satisfaction, and the cost-benefit breakdown.

#### Physiology and Function of Swallowing

##### Safety of the Swallowing Process

Swallowing safety mainly involves the safety of swallowing in the pharyngeal phase (eg, to prevent aspiration, penetration, pharyngeal residue, and aspiration pneumonia). Swallowing safety mainly involves the safety of swallowing in the pharyngeal phase. Swallowing in the pharyngeal phase must be safe and effective. To ensure the safety and effectiveness of swallowing after treatment, it is necessary to train the tongue, soft palate, pharyngeal wall, laryngeal muscle, thyroid cartilage, and upper esophageal sphincter to coordinate and complete swallowing. In 8% (1/13) of the studies, preventive swallowing training and progressive resistance training (ie, dual-mode rehabilitation training) were carried out during radiotherapy. The authors compared the effects of remote swallowing training mode and conventional rehabilitation after intervention. The results showed no significant differences in aspiration scores between patients with HNC in the intervention and control groups when swallowing liquids and semisolids. However, a significant difference was found in the pharyngeal residual (ie, significantly reduced in the intervention group) after 2 months of intervention [19]. Starmer et al [18] found that, although there was no significant difference in swallowing safety between the experimental and control groups after 7 weeks of a home-based diversity swallowing training combination program for patients with HNC, there was a significantly greater improvement in dysphagia in the experimental group than in the control group. Another asynchronous remote swallowing intervention based on WeChat included scientific and standardized rehabilitation training measures and active treatment, and it was found to improve the chewing and swallowing functions of patients with oral and oropharyngeal tumors within 6 months after surgery. The results also showed that the effective remote rehabilitation intervention could save medical resources and reduce personnel contact. It can significantly improve the long-term (3-6 months) training effect [20].

Another study compared the effects of synchronous and asynchronous remote swallowing rehabilitation. The effects of remote swallowing rehabilitation after independent training intervention were also compared. Swallowing safety was determined using the VFSS, MBS, and PAS. Face-to-face treatment and the *SwallowIT* application intervention had the same effects in terms of reduced occurrence of aspiration when swallowing food of different consistencies [29].

##### Swallowing Muscle Activity

In 31% (4/13) of the studies, the muscle groups in the maxillofacial region that participate in mouth opening and chewing were gradually relaxed, and the fibrotic muscles were slowly stretched through mouth opening training. However, even after training, the maximum mouth opening of patients with dysphagia was still decreased with increased time since treatment [24,25,29]. A tailored swallowing rehabilitation program achieved better mouth opening in the intervention group [19]. Another study reported a significant correlation between mouth opening exercises and motor performance, namely, that experiencing more mouth opening difficulties in the previous week reduced the likelihood of successful high-intensity motor performance in the middle of the following week [25].

In total, 8% (1/13) of the studies used the mobile health swallowing system equipped with sensors and surface electromyography biofeedback devices as swallowing treatment for patients with dysphagia after HNC treatment; however, it remained unclear which exercise more effectively activated the related swallowing muscles and increased the muscle movement amplitude [23].

##### Oral Feeding Safety

Oral feeding ability is an important sign of recovery of swallowing function. The guided self-help swallowing intervention by Jansen et al [21] achieved significantly improved swallowing function in patients in the remote intervention group compared with those in the control group over time. In particular, the differences in duration and oral feeding at the 6-month time point were statistically significant [21]. One study showed no significant difference in oral feeding function (evaluated using the Functional Oral Intake Scale) between the intervention and control groups; however, tube feeding dependence rates were reduced in patients in the remote rehabilitation group who adhered to the swallowing training plan [19]. Another study also found that participants in remote swallowing rehabilitation during HNC treatment had better dietary outcomes [18].

In a study comparing asynchronous and synchronous telemedicine, Wall et al [29] showed no significant differences between remote swallowing intervention and face-to-face treatment with speech pathologists in restoring oral feeding and gavage dependence.

#### Nutritional Status

Swallowing interventions can reduce the complications of malnutrition caused by delayed oral feeding. The intervention by Hajdú et al [19] provided swallowing practice and drew attention to how to maintain oral diet. Nutritional counseling, provided through the collaboration of dieticians and speech pathologists, focused on maintaining an adequate oral diet in terms of nutritional levels and prevention of weight loss. The results showed similar recovery effects in body weight and nutritional scores between patients receiving remote rehabilitation and those receiving active in-hospital rehabilitation [19].

#### Swallowing-Related Quality of Life

Patients attach particular importance to quality of life related to dysphagia, and multiple studies have also shown that the quality of life related to swallowing function, physical functioning, and social functioning was significantly higher after home telerehabilitation in patients with HNC [18,19,21,23,31]. One study explored the relationship between participants’ motor performance and swallowing-related quality of life. The results showed that there was no significant difference in the quality of life among low-, medium-, or high-intensity training groups after 6- or 12-week interventions, but the quality of life of participants who underwent low- or medium-intensity training was slightly higher than that of participants who underwent high-intensity training [25].

#### Swallowing-Related Negative Emotions

Wall et al [29] provided personal health management guidance for patients with HNC according to their characteristics and individual needs. The results showed that the Hospital Anxiety and Depression Scale scores of the face-to-face intervention group decreased from baseline to after treatment, whereas those of the *SwallowIT* application group did not change significantly at 6 weeks and 3 months after radiotherapy [29].

#### Adherence to Telerehabilitation Exercise

Telerehabilitation has great potential to promote patient self-management and improve adherence. The adherence rates varied widely among all the included studies, from 27% to 81.8%.

In total, 38% (5/13) of the studies showed that adherence to rehabilitation interventions tended to decrease as the intervention time increased. Cnossen et al [25] reported that participant adherence to remote rehabilitation decreased from 70% at week 6 to 38% at week 12. Constantinescu et al [23] investigated adherence in the first and sixth weeks of the intervention and found that it decreased from 83.5% to 77.5%. Starmer et al [18] found that, regardless of the treatment group, adherence decreased over the course of radiation therapy, with the best adherence reported during the first 2 weeks. In 15% (2/13) of the studies comparing asynchronous and synchronous telemedicine, Wall et al [29] showed greater adherence at 1 to 3 weeks of chemotherapy or radiotherapy than at 4 to 6 weeks. Baudelet et al [28] found that adherence rates in the therapist, paper, and app groups were lower at 3 and 4 weeks than at 1 and 2 weeks after HNC treatment. Moreover, Wall et al [29] showed that adherence was higher in the remote intervention group than in the patient guidance group; however, Baudelet et al [28] found that adherence in the app group was significantly lower than that in the paper group at weeks 3 and 4.

A total of 15% (2/13) of the studies investigated adherence with low-, medium-, and high-intensity rehabilitation training; adherence with low-intensity training was high, whereas adherence with medium- and high-intensity training was low [24,25].

#### Participants’ Satisfaction With Telerehabilitation

The functions of web-based and offline interaction and the rich audio-video knowledge base of telerehabilitation change the traditional intervention mode, improve patients’ sense of experience, and meet the needs of patients’ health education. A total of 38% (5/13) of the studies evaluated participants’ satisfaction with swallowing rehabilitation interventions. The results all indicated that remote interventions had higher satisfaction rates and that participants supported remote medical treatment. The results of 15% (2/13) of the studies showed that participants were highly satisfied with and supportive of the therapeutic effects, treatment methods, and suggestions of telemedicine services. Participants believed that rehabilitation training improved their motivation and enabled them to cope with treatment better and faster. After training, participants reported higher satisfaction because of social and emotional support [19,27,32]. One of the studies also surveyed the satisfaction of nursing staff and clinicians with telemedicine services; the use of telemedicine not only allowed for adequate clinical evaluation but also effectively addressed the clinical problems of patients [27]. In total, 15% (2/13) of the studies comparing asynchronous and synchronous telemedicine investigated service provision preferences and showed contrasting results. Wall et al [29] found that most participants preferred face-to-face and remote rehabilitation instruction. However, Baudelet et al [28] found that participants’ acceptance of remote applications was not high.

#### Cost and Cost-Effectiveness of Telerehabilitation

Compared with traditional face-to-face follow-up, home telerehabilitation is a more efficient and low-cost approach. A study summarizing the cost and cost-effectiveness of home-based telerehabilitation in the Netherlands found that the probability of remote interventions being cost-effective was 57% to 70%. At present, the cost of intervention is relatively high because of its limitations and the low number of potential users. A cost-benefit analysis at the 6-month follow-up showed that, although the total cost for patients in the intervention group was €232 (US $255.53) higher than that for patients in the control group, the rehabilitative effect was significantly greater for those in the intervention group [22]. Therefore, the included studies indicate that remote interventions reduce patient costs or result in greater rehabilitative effects than in-person interventions.

### Telerehabilitation Influencing Factors

This section describes the factors influencing home-based swallowing telerehabilitation.

The supporting factors of home telerehabilitation can relieve patients’ anxiety, fear, and depression; improve disease awareness; reduce disease uncertainty; and improve satisfaction with participating in treatment decision-making, thereby improving the effect of rehabilitation therapy and long-term health-related quality of life. Textbox 2 shows the specific predictors examined herein.

**Barriers to and facilitators of home-based telerehabilitation.**

**Sociodemographic characteristics**

- Sex, age, race, marital status, children, family support, caregiver support, and educational attainment

**Clinical characteristics**

- Type of cancer, mode of treatment (multimodal treatment with chemoradiotherapy or surgery), stage of disease, side effects of radiotherapy (oral mucositis, skin and oral wounds, and oral infections), and tumor complications

**Physiological functioning**

- Dysphagia, limited mouth opening, gavage dependence, symptoms (pain, nausea, fatigue, and neck and shoulder swelling or stiffness), physical fitness, and BMI

**Behavior**

- Motor perception, motivation, confidence, motor motivation, motor history, social ability, self-management, emotional problems (anxiety, depression, worry, fear, and panic attacks), smoking status, and drinking status

**Technology**

- Application or internet installation issues, reasonable design, software speed, operability, cost, privacy and data security, ease of use, ease of understanding, and ease of adherence

**Rehabilitation processes**

- Swallowing training type, duration of a single intervention session, intervention intensity, intervention frequency, and swallowing safety (aspiration, swallowing error, penetration, cough, and pharyngeal residue)

**Educational content**

- Information, illustrations and videos for swallowing exercises, principles of swallowing exercises, nutrition or diet, recording videos, tumor self-examination and recurrence, oral self-management, fatigue and energy management, and survival care plan

## Discussion

### Principal Findings

Remote dysphagia management is paramount for patients with HNC, family caregivers, health care providers, and health service policy makers. The effective use of home-based telemedicine interventions for dysphagia was a major concern during the COVID-19 pandemic. Our systematic review of 13 studies on home-based telerehabilitation dysphagia treatment provides an up-to-date review of the field, describing the safety, effectiveness, quality of life, and adherence regarding telerehabilitation for patients with swallowing disorders from HNC and identifying various facilitators and obstacle elements related to telerehabilitation. Owing to the limited number of studies that met the inclusion criteria, the study designs, and the methodological limitations of inconsistent measurements of results, we cannot definitively conclude that remote rehabilitation has a positive impact on patients with HNC with dysphagia. However, this systematic review provides evidence on the subject.

This study indicated that home-based remote rehabilitation of patients with HNC with dysphagia may reduce the safety risks of swallowing and oral feeding, reduce malnutrition and the decline in quality of life because of delayed oral feeding, and improve the (previously low) adherence rates of rehabilitation training. Moreover, tailored rehabilitation, information provision, and behavior change strategies appeared more effective in treating dysphagia, and more pronounced improvements in swallowing and physiological function may be observed over time.

### Advantages and Effects of Swallowing Telerehabilitation in Patients With HNC

Remote swallowing rehabilitation is a new mode of rehabilitation led by intelligent remote equipment and flexible participation of therapists. According to the swallowing function of patients with HNC, web-based evaluation, and push rehabilitation guidance suggestions, it supervises the rehabilitation process; records the rehabilitation data; and builds a communication, feedback, and follow-up platform among patients, their families, and therapists. For patients, the use of telerehabilitation time is flexible, free, and convenient for family supervision to ensure that the scheduled swallowing training movements are accurately repeated, reduce injury and human error, avoid the delay of treatment caused by inconvenient travel and regional restrictions, and reduce the economic burden. For rehabilitation therapists, remote rehabilitation can be realized from “one to one” to “one to many,” and it can also make more patients participate, thereby facilitating the collection of follow-up data.

This study shows that home-based remote swallowing rehabilitation therapy can effectively improve the swallowing and dietary function of patients with HNC and support their nursing and psychological needs. Notably, all studies in this review were based on the remote treatment mode of asynchronous telemedicine. Although there is a certain gap between the remote treatment mode and the “one-to-one” rehabilitation mode of therapists, there was no significant difference between the treatment effect and conventional rehabilitation treatment. However, whether remote swallowing rehabilitation can be an effective alternative to traditional rehabilitation in clinical practice needs to be evaluated in the long term, and future research still needs to continue to improve remote rehabilitation.

The Hajdú et al [19] RCT of home-based swallowing rehabilitation training showed a modest effect on reducing pharyngeal residue but no effect of this intervention on swallowing safety in patients with HNC regarding penetration and aspiration. The reason for the absence of a greater impact on aspiration is largely related to the short duration of the intervention and the small real differences between groups [19]. One study showed that remote rehabilitation training involving only low- to moderate-intensity training for symptom-oriented exercise had a positive impact on physiological function and quality of life [33]. Another study highlighted that low-intensity regimens may be easier to achieve and are effective at motivating patients, improving adherence rates, and achieving desired clinical outcomes [29]. This is consistent with the findings of Langmore et al [34], who found that practicing the simple act of swallowing improved patients’ skills, ease of use, and eating speed, helping them swallow more challenging foods safely and effectively. Furthermore, in older patients, there may be problems with decreased physical function, reduced endurance, and a lower tolerance to the training load. Therefore, we recommend that a low-intensity training regimen may be more suitable to their specific needs and reduce the risk of potential physical discomfort or excessive fatigue. Therefore, the type of home-based rehabilitation training selected is important and should be combined with the appropriate training intensity and duration to achieve the desired intervention effect. Future research should design tailored swallowing plans through training that caters to patients’ abilities and interests, finding a balance among training intensity, training duration, and treatment adherence.

Improved swallowing function is essential to restore oral feeding. The importance of combining dietary intake and swallowing training was supported by 15% (2/13) of the studies, in which maintaining an oral diet during multimodal therapy was a powerful predictor of long-term functional swallowing [19,35]. Another study also reported successful maintenance of the nutritional status of patients through home-based telemedicine services that combined swallowing management and dietary safety [27]. Moreover, safe oral feeding and swallowing of dilute fluids is a challenge for patients with dysphagia. Bolus adjustment (including thickening fluids) is usually performed to help the patient eat safely and orally to satisfy nutritional needs, but it is important to ensure that excessive adjustment of food consistency does not result in increased pharyngeal residue after swallowing [36]. Hence, rehabilitation dietitians and speech pathologists need to cooperate to design personalized nutritional recipes for remote push during home-based rehabilitation. However, owing to the complexity of bolus adjustment in the clinical management of dysphagia, multidisciplinary planning and careful clinical decision-making are needed to determine effectiveness and safety.

In the process of swallowing function diagnosis and treatment, patients with HNC need timely, long-term, and strong health information support and guidance. Comprehensive, accurate, and systematic assessment of the health information needs of patients with HNC is the first step toward providing individualized and targeted professional information support. It is also an important basis for constructing information support strategies based on the real needs of patients. Home-based telerehabilitation enables patients to receive services that provide higher motivation, informational content, support, and encouragement [37] and to improve their quality of life. The results of this study show that remote rehabilitation interventions can improve the quality of life of patients with HNC with dysphagia compared with standard rehabilitation and routine care, which is consistent with a previous study [38]. Studies have found that patients with higher levels of educational attainment, family income, and professional ability are more likely to perceive the potential benefits of rehabilitation training on their quality of life [39], which suggests that more attention should be paid to patients with lower levels of educational attainment, family income, and occupation. Future research needs to deeply explore the specific content and reasons for the information needs of different patients with HNC through qualitative interviews to optimize the telerehabilitation information support system. In addition, studies have shown that more than half (58%) of the HNC dysphagia caregiver population experiences clinically significant levels of psychosocial distress during or after treatment, especially during mealtimes and chemoradiation therapy, which may have a significant impact on caregivers’ health and quality of life [40]. Therefore, telemedicine will also be necessary in the future to explore ways of supporting caregivers at specific time points during patient rehabilitation therapy to counteract caregiver crises and negative psychosocial outcomes.

Synchronous telemedicine also plays an important role in planning and implementing dysphagia treatment and involves incorporating the underlying etiology based on swallowing anatomy or physiological damage obtained through a comprehensive clinical or instrumental assessment (eg, VFSS, MBS, or FEES) and making decisions regarding personalized swallowing and oral dysphagia management. For example, there is the absence of one factor in swallowing and the compensation for another factor to achieve safe and effective swallowing. Remote VFSS assessments have been repeatedly demonstrated to be safe, effective, and reliable compared with traditional on-site swallowing assessments [32,41]. Studies focusing on synchronous telemedicine were not included in this review, but they also need to be considered. Future research should combine synchronous care, which not only monitors patient training but also includes clinical and procedural systems to support real-time contrast-swallowing remote practice.

### Adherence Management and Factors Influencing Swallowing Telerehabilitation in Patients With HNC

HNC, as a complex chronic disease, urgently requires the self-monitoring of disease changes, changes in unhealthy lifestyles, and rehabilitation exercises after chemotherapy or radiotherapy. Increasing patients’ adherence and supporting patients’ self-management are of great significance for improving patients’ health outcomes, improving quality of life, and reducing medical costs. The results of this study showed that, although the swallowing rehabilitation adherence in patients with HNC with telerehabilitation was improved compared with standard rehabilitation, it also decreased significantly with the treatment time, indicating that patients still lack the necessary confidence or skills to actively manage the disease. The reasons are analyzed as follows. First, influenced by the traditional medical concept, most patients are still overdependent on medical staff after discharge. Second, the chronic side effects caused by radiotherapy, such as cancer-related fatigue, pain, difficulty in opening the mouth, and head and neck fibrosis, were gradually aggravated, and the patients’ knowledge of disease treatment and nursing was insufficient.

This study aimed to identify methods for improving adherence to telerehabilitation. It is recommended to design a tailor-made telemedicine service to solve personalized dysphagia functional problems and address health-related risk behaviors and psychological problems (eg, depression) to ensure adherence to rehabilitation [42] and meet the specific needs of patients with HNC.

An interesting comparison of synchronous and asynchronous telemedicine showed that the app group had the lowest adherence rate of the 3 service models. The reason is that the elements in the app were not compelling enough and the design was not convenient enough. Therefore, the perceived usefulness and comfort of the website or app are important factors affecting participants’ acceptance of and adherence to home-based telerehabilitation and were also found to be positive supportive factors for older people to receive home-based telemedicine services [43].

Age, gender cognition, motivation, physical mobility, and sensory and perceptual function are important factors influencing remote swallowing rehabilitation. For older individuals, the ability to understand and use is limited. When individualizing the rehabilitation program, it is necessary to further confirm the practicality of the intervention and design an appropriate application program interface and function and consider font and color contrast or sensitivity, among other things. Younger patients placed more emphasis on the social and emotional domains (employment, functioning, and postrehabilitation psychology) than older patients [42]. As the onset age of patients with HNC decreases, future research should explore the information and emotional needs of younger patients. Furthermore, women need more emotional and psychosocial support than men and are more willing to receive information to deal with related stress and anxiety [44]. Patients with newly diagnosed HNC pay more attention to the medical field than patients with recurrent disease [45] as patients with recurrent disease have already received information and experienced the disease; thus, they need more emotional support. Hence, remote interventions (eg, websites and applications) should also be configured to provide education, feedback, information, communication, and support that meet the needs of different populations and stages of dysphagia to improve overall patient adherence to telerehabilitation.

Studies have emphasized the importance of biofeedback and social engagement. When biofeedback is combined with swallowing training, feedback, or education, it may lead to enhanced improvements in patient adherence [46,47]. This is due to the regulation of patients’ bad emotions and behaviors through incentive strategies during exercise and as a mediating variable to predict patient compliance. Feeding training data into the system is also a way of alerting and tracking when biofeedback is not being used. Leaderboards and status sharing are important elements of social engagement in many other health applications, but studies have shown that performance comparisons between participants can lead to reduced self-efficacy [48]; however, social engagement can also take the form of anonymous incentivized information exchange between participants. In addition, the self-reported experiences of participants who recovered swallowing function inspired patients to adhere to home-based telerehabilitation.

Therefore, future high-quality research can identify motivations for participants to persist in rehabilitation according to age, sex, disease status (new diagnosis or recurrence), treatment duration, radiotherapy duration, radiotherapy side effects, swallowing practice demonstration, and other aspects using websites or applications and provide information for personalized dysphagia management and support. According to the survey results of this study, we recommend that swallowing function should first be assessed through face-to-face or synchronous telemedicine (VFSS, MBS, and FEES) and swallowing training suitable for patients (such as strengthening hyoid muscle group training) should be formulated according to the assessment results. It is then recommended to build a synchronous and asynchronous home-based rehabilitation mode. The main functions are intelligent questions and answers (providing health education videos and text replies), light consultations, health education video columns, remote audio- and videoconference, and manual appointment services. As for rehabilitation training, it is recommended that preventive low-intensity training targeting the muscles involved in swallowing injury be carried out before rehabilitation therapy and continue for 3 months after the end of treatment. During this period, speech therapists regularly assess swallowing function, make individualized adjustments, and guide intervention. Moreover, in the design of remote intervention applications, combining the gamification of exercise, robot equipment, virtual reality equipment, biofeedback systems, smart home systems, and social engagement can motivate patients to actively complete rehabilitation training and create a virtuous cycle that improves functionality and adherence to rehabilitation. However, rehabilitation technology is still in the initial stage of development, and further research on the effectiveness and safety of these rehabilitation technologies for the population with HNC is needed.

### Strengths and Limitations

Our systematic review comprehensively examined the impact of home-based teleintervention in the population with HNC dysphagia as well as the evidence for facilitators of favorable outcomes. It summarized the safety, effectiveness, and adherence regarding remote interventions in patients with HNC with dysphagia as well as the related barriers and facilitators. However, we acknowledge that our systematic review has some limitations. First, although 2 researchers separately conducted the literature search and extraction, we admit that it is possible that we overlooked some relevant studies. Second, the form of home-based remote intervention used in the included studies was mainly asynchronous telemedicine rather than synchronous telemedicine; in addition, the intervention content, training duration, training intensity, and evaluation indicators of the family-based remote interventions used in the studies differed. This heterogeneity among studies is a general limitation of other systems used to evaluate swallowing rehabilitation. In addition, not all the studies reported swallowing clinical findings and cost follow-up data; the studies mostly focused on the feasibility and implementation of remote interventions, which may have led to the limited impact reflected on the population with HNC dysphagia.

### Implications for Practice and Research

#### Health Care System and Social Support

By demonstrating the effectiveness of telerehabilitation interventions, this study can inform public policy development and reduce current barriers to better support the population with HNC.

#### Collaboration Between Speech Pathologists, Patients With Dysphagia, and Technical Developers

Rapid clinical transformation of technological innovation can be achieved through cross-disciplinary and cross-clinical cooperation and targeted remote rehabilitation systems (in terms of age, sex, dysphagia etiology, comorbidities, physical health, and other factors). These can be tailored to different populations; swallowing training can be adjusted to suit individual needs; and training (in terms of frequency, intensity, and duration) can be formulated to suit the current motor ability, performance, and preferences of individuals. Interactive internet-based feedback improved patient self-management and rehabilitation adherence, and educational resources and training samples met the information needs of patients with HNC and their caregivers, thereby maximizing the effectiveness and acceptability of remote swallowing rehabilitation.

#### Recommendations for Future Studies

High-quality, systematic, rigorously designed, randomized multicenter studies are needed to assess the rehabilitation effects of different or combined remote rehabilitation methods in patients with HNC and explore the balance among training duration, effectiveness, and adherence to provide stronger evidence in support of remote intervention. In addition, qualitative interviews or longitudinal follow-up can be conducted in the future, focusing on interviewing and documenting the practical problems and experiences of different patients to study adherence management and its influencing factors in depth and focusing on patient self-efficacy, educational information, behavioral skills, and psychosocial rehabilitation, including the caregivers of patients, to promote the development of telemedicine.

### Conclusions

Home-based telerehabilitation has shown great potential in reducing the safety risks of swallowing and oral feeding, improving quality of life and adherence, and meeting the information needs for dysphagia in patients with HNC. The lack of standardization across methods and metrics limits our understanding. Therefore, future research needs to deeply explore the intervention effects of different remote interventions in the prevention of dysphagia in patients with HNC; adopt tailored telemedicine interventions (eg, multidomain intervention content, mode, type, frequency, duration, and information needs); and increase sample sizes to implement long-term intervention studies, achieve optimal recovery, and maximize adherence to home-based training programs.

## Appendix

Multimedia Appendix 1 Search terms and methodological quality.

Multimedia Appendix 2 Included studies and interventions.

## Abbreviations

EORTC: European Organisation for Research and Treatment of Cancer
FEES: fiberoptic endoscopic evaluation of swallowing
HNC: head and neck cancer
MBS: modified barium swallow study
PAS: penetration-aspiration scale
PRISMA: Preferred Reporting Items for Systematic Reviews and Meta-Analyses
RCT: randomized controlled trial
VFSS: videofluoroscopic swallow study
