# Supplementary material for: Effectiveness of Home-Based Telerehabilitation Interventions for Dysphagia in Patients With Head and Neck Cancer: Systematic Review
Source: J Med Internet Res. 2023 Sep 8;25:e47324. doi: 10.2196/47324 (PMC10517384; doi:10.2196/47324)
Supplement: Multimedia Appendix 1 [file jmir_v25i1e47324_app1.docx]

**Search terms**

**Database: PubMed**

#1 "Head and Neck Neoplasms" [Mesh Terms]

#2 "Neoplasms, Head and Neck" [Title/Abstract]

#3 "Head, Neck Neoplasms" [Title/Abstract]

#4 "Cancer of Head and Neck" [Title/Abstract]

#5 "Head and Neck Cancer" [Title/Abstract]

#6 "Cancer of the Head and Neck" [Title/Abstract]

#7 "Upper Aerodigestive Tract Neoplasms" [Title/Abstract]

#8 "UADT Neoplasms" [Title/Abstract]

#9 "Neoplasm, UADT" [Title/Abstract]

#10 "Neoplasms, UADT" [Title/Abstract]

#11 "UADT Neoplasm" [Title/Abstract]

#12 "Neoplasms, Upper Aerodigestive Tract" [Title/Abstract]

#13 "Head Neoplasms" [Title/Abstract]

#14 "Neoplasms, Head" [Title/Abstract]

#15 "Neck Neoplasms" [Title/Abstract]

#16 "Neoplasms, Neck" [Title/Abstract]

#17 "Cancer of Head" [Title/Abstract]

#18 "Head Cancer" [Title/Abstract]

#19 "Cancer of the Head" [Title/Abstract]

#20 "Cancer of Neck" [Title/Abstract]

#21 "Neck Cancer" [Title/Abstract]

#22 "Cancer of the Neck" [Title/Abstract]

#23 OR #1-#22

#24 "Mouth Neoplasms" [Mesh Terms]

#25 "Mouth Neoplasm" [Title/Abstract]

#26 "Neoplasm, Mouth" [Title/Abstract]

#27 "Neoplasms, Oral" [Title/Abstract]

#28 "Neoplasm, Oral" [Title/Abstract]

#29 "Oral Neoplasm" [Title/Abstract]

#30 "Oral Neoplasms" [Title/Abstract]

#31 "Neoplasms, Mouth" [Title/Abstract]

#32 "Cancer of Mouth" [Title/Abstract]

#33 "Mouth Cancer" [Title/Abstract]

#34 "Oral Cancer" [Title/Abstract]

#35 "Cancer, Oral" [Title/Abstract]

#36 "Cancers, Oral" [Title/Abstract]

#37 "Oral Cancers" [Title/Abstract]

#38 "Cancer of the Mouth" [Title/Abstract]

#39 "Mouth Cancer" [Title/Abstract]

#40 "Cancer, Mouth" [Title/Abstract]

#41 "Cancers, Mouth" [Title/Abstract]

#42 OR #24-#41

#43 "Otorhinolaryngologic Neoplasms"[Mesh Terms]

#44 "Neoplasm, Otorhinolaryngologic"[Title/Abstract]

#45 "Otorhinolaryngologic Neoplasm"[Title/Abstract]

#46 "Ootorhinolaryngeal Neoplasms"[Title/Abstract]

#47 "Neoplasm, Ootorhinolaryngeal"[Title/Abstract]

#48 "Neoplasms, Ootorhinolaryngeal"[Title/Abstract]

#49 "Ootorhinolaryngeal Neoplasm"[Title/Abstract]

#50 "Neoplasms, Otorhinolaryngological"[Title/Abstract]

#51 "Neoplasm, Otorhinolaryngological"[Title/Abstract]

#52 "Otorhinolaryngological Neoplasm"[Title/Abstract]

#53 "Otorhinolaryngological Neoplasms"[Title/Abstract]

#54 "Neoplasms, Otorhinolaryngologic"[Title/Abstract]

#55 "Otorhinolaryngeal Cancer"[Title/Abstract]

#56 "Cancer, Otorhinolaryngeal"[Title/Abstract]

#57 "Cancers, Otorhinolaryngeal"[Title/Abstract]

#58 "Otorhinolaryngeal Cancers"[Title/Abstract]

#59 OR #43-#58

#61 "Hypopharyngeal Neoplasms" [Mesh Terms]

#62 "Hypopharyngeal Neoplasm" [Title/Abstract]

#63 "Neoplasm, Hypopharyngeal" [Title/Abstract]

#64 "Neoplasms, Hypopharyngeal" [Title/Abstract]

#65 "Hypopharyngeal Cancer" [Title/Abstract]

#66 "Cancer, Hypopharyngeal" [Title/Abstract]

#67 "Cancers, Hypopharyngeal" [Title/Abstract]

#68 "Hypopharyngeal Cancers" [Title/Abstract]

#69 OR #61-#68

#70 "Laryngeal Neoplasms" [Mesh Terms]

#71 "Laryngeal Neoplasm" [Title/Abstract]

#72 "Neoplasms, Laryngeal" [Title/Abstract]

#73 "Laryngeal Neoplasm" [Title/Abstract]

#74 "Neoplasm, Laryngeal" [Title/Abstract]

#75 "Larynx Neoplasms" [Title/Abstract]

#76 "Larynx Neoplasm" [Title/Abstract]

#77 "Neoplasm, Larynx" [Title/Abstract]

#78 "Neoplasms, Larynx" [Title/Abstract]

#79 "Cancer of Larynx" [Title/Abstract]

#80 "Larynx Cancers" [Title/Abstract]

#81 "Laryngeal Cancer" [Title/Abstract]

#82 "Cancer, Laryngeal" [Title/Abstract]

#83 "Cancers, Laryngeal" [Title/Abstract]

#84 "Laryngeal Cancers" [Title/Abstract]

#85 "Larynx Cancer" [Title/Abstract]

#86 "Cancer, Larynx" [Title/Abstract]

#87 "Cancers, Larynx" [Title/Abstract]

#88 "Cancer of the Larynx" [Title/Abstract]

#89 OR #70-#88

#90 "Nasopharyngeal Neoplasms" [Mesh Terms]

#91 "Nasopharyngeal Neoplasm" [Title/Abstract]

#92 "Neoplasm, Nasopharyngeal" [Title/Abstract]

#93 "Neoplasms, Nasopharyngeal" [Title/Abstract]

#94 "Nasopharynx Neoplasms" [Title/Abstract]

#95 "Nasopharynx Neoplasm" [Title/Abstract]

#96 "Neoplasm, Nasopharynx" [Title/Abstract]

#97 "Neoplasms, Nasopharynx" [Title/Abstract]

#98 "Cancer of Nasopharynx" [Title/Abstract]

#99 "Nasopharynx Cancers" [Title/Abstract]

#100 "Nasopharyngeal Cancer" [Title/Abstract]

#101 "Cancer, Nasopharyngeal" [Title/Abstract]

#102 "Cancers, Nasopharyngeal" [Title/Abstract]

#103 "Nasopharyngeal Cancer" [Title/Abstract]

#104 "Nasopharynx Cancer" [Title/Abstract]

#105 "Cancer, Nasopharynx" [Title/Abstract]

#106 "Cancers, Nasopharynx" [Title/Abstract]

#107 "Cancer of the Nasopharynx" [Title/Abstract]

#108 OR #90-#107

#109 HNC OR HNSCC OR SCCHN OR OPSCC

#110 OR #23 OR #42 OR #59 OR #69 OR #89 OR #108 OR #109

#111 "Deglutition Disorders"[Mesh Terms]

#112 "Deglutition Disorder" [Title/Abstract]

#113 "Disorders, Deglutition" [Title/Abstract]

#114 "Swallowing Disorders" [Title/Abstract]

#115 "Swallowing Disorder" [Title/Abstract]

#116 "Dysphagia" [Title/Abstract]

#117 "Oropharyngeal Dysphagia" [Title/Abstract]

#118 "Dysphagia, Oropharyngeal" [Title/Abstract]

#119 "Esophageal Dysphagia" [Title/Abstract]

#120 "Dysphagia, Esophageal" [Title/Abstract]

#121 OR #111-#120

#122 "Telemedicine" [Title/Abstract]

#123 "Mobile Health" [Title/Abstract]

#124 "Health, Mobile" [Title/Abstract]

#125 "mHealth" [Title/Abstract]

#126 "Telehealth" [Title/Abstract]

#127 "eHealth" [Title/Abstract]

#128 "Telerehabilitation" [Title/Abstract]

#129 "Telerehabilitations" [Title/Abstract]

#130 "Tele-rehabilitation" [Title/Abstract]

#131 "Tele rehabilitation" [Title/Abstract]

#132 "Tele-rehabilitations" [Title/Abstract]

#133 "Remote Rehabilitation" [Title/Abstract]

#134 "Rehabilitation, Remote" [Title/Abstract]

#135 "Rehabilitations, Remote" [Title/Abstract]

#136 "Remote Rehabilitations" [Title/Abstract]

#137 "Virtual Rehabilitation" [Title/Abstract]

#138 "Rehabilitation, Virtual" [Title/Abstract]

#139 "Rehabilitations, Virtual" [Title/Abstract]

#140 "Virtual Rehabilitations" [Title/Abstract]

#141 "Telecare" [Title/Abstract]

#142 "Telehomecare" [Title/Abstract]

#143 "Mcare" [Title/Abstract]

#144 "M-care" [Title/Abstract]

#145 "Telemonitor" [Title/Abstract]

#146 "Teletherapy" [Title/Abstract]

#147 "Teleconsultation" [Title/Abstract]

#148 "Remote consultation" [Title/Abstract]

#149 "Remote supervision" [Title/Abstract]

#150 "Remote monitoring" [Title/Abstract]

#151 "Remote evaluation" [Title/Abstract]

#152 "Telecommunication" [Title/Abstract]

#153 "Telemanagement" [Title/Abstract]

#154 "Internet-based" [Title/Abstract]

#155 "Televideo" [Title/Abstract]

#156 "Interactive video" [Title/Abstract]

#157 "Virtual Reality Exposure Therapy" [Title/Abstract]

#158 "Virtual Reality Therapy" [Title/Abstract]

#159 "Reality Therapies, Virtual" [Title/Abstract]

#160 "Reality Therapy, Virtual" [Title/Abstract]

#161 "Therapies, Virtual Reality" [Title/Abstract]

#162 "Therapy, Virtual Reality" [Title/Abstract]

#163 "Virtual Reality Therapies" [Title/Abstract]

#164 OR #122-#163

#165 #110 AND #121 AND #164

**Database: Embase**

#1 'head and neck tumor'/exp

#2 'ear nose throat tumor':ab,ti

#3 'ear nose throat tumour':ab,ti

#4 'ENT tumor':ab,ti

#5 'ENT tumour':ab,ti

#6 'head and neck neoplasms':ab,ti

#7 'head and neck tumour':ab,ti

#8 'head neck tumor':ab,ti

#9 'head neck tumour':ab,ti

#10 'ORL tumor':ab,ti

#11 'ORL tumour':ab,ti

#12 'otorhinolaryngeal tumor':ab,ti

#13 'otorhinolaryngeal tumour':ab,ti

#14 'otorhinolaryngologic neoplasms':ab,ti

#15 'otorhinolaryngologic tumor':ab,ti

#16 'otorhinolaryngologic tumour':ab,ti

#17 'otorhinolaryngological tumor':ab,ti

#18 'otorhinolaryngological tumour':ab,ti

#19 'tumor, head and neck':ab,ti

#20 'tumour, head and neck':ab,ti

#21 HNC:ab,ti

#22 HNSCC:ab,ti

#23 SCCHN:ab,ti

#24 OPSCC:ab,ti

#25 or #1-#24

#26 'dysphagia'/exp

#27 'Aphagopraxia':ab,ti

#28 'deglutition difficulty':ab,ti

#29 'deglutition disorder':ab,ti

#30 'deglutition disorders':ab,ti

#31 'difficult deglutition':ab,ti

#32 'difficulty in swallowing':ab,ti

#33 'difficulty swallowing':ab,ti

#34 'dysphagias':ab,ti

#35 'swallowing difficult':ab,ti

#36 'swallowing difficultness':ab,ti

#37 'swallowing difficulty':ab,ti

#38 'swallowing disorder':ab,ti

#39 or #26-#38

#40 'swallowing'/exp

#41 'deglutition':ab,ti

#42 'oropharyngeal swallow':ab,ti

#43 'reflex, deglutition':ab,ti

#44 'swallow (deglutition) ':ab,ti

#45 'swallow (ingestion) ':ab,ti

#46 'swallow function':ab,ti

#47 'swallow reflex':ab,ti

#48 'swallowing reflex':ab,ti

#49 or #40-#48

#50 'Motor activity'/exp

#51 'Strength training':ab,ti

#52 or #50-#51

#53 'Exercise"/exp

#54 'Exercise training':ab,ti

#55 or #53-#54

#56 'Kinesiotherapy'/exp

#57 'Exercise therapy':ab,ti

#58 'Exercise program':ab,ti

#59 'Physical exercise':ab,ti

#60 or #56-#59

#61 'Occupational therapy'/exp

#62 'Exercise intervention':ab,ti

#63 'Physical activity':ab,ti

#64 or #61-#63

#65 'Pre-treatment swallowing intervention':ab,ti

#66 'Prophylactic swallowing therapy':ab,ti

#67 'Prophylactic swallowing rehabilication':ab,ti

#68 'Prophylactic swallowing exercises':ab,ti

#69 'Pre-treatment care':ab,ti

#70 or #65-#69

#71 'rehabilitation'/exp

#72 'functional readaptation':ab,ti

#73 'medical rehabilitation':ab,ti

#74 'readaption':ab,ti

#75 'readjustment':ab,ti

#76 'rehabilitation concept':ab,ti

#77 'rehabilitation engineering':ab,ti

#78 'rehabilitation potential':ab,ti

#79 'rehabilitation process':ab,ti

#80 'rehabilitation program':ab,ti

#81 'rehabilitation programme':ab,ti

#82 'rehabilitation, medical':ab,ti

#83 'rehabilitative treatment':ab,ti

#84 'resocialization':ab,ti

#85 'resocialisation therapy':ab,ti

#86 'resocialization':ab,ti

#87 'resocialization therapy':ab,ti

#88 'revalidation':ab,ti

#89 or #71-#88

#90 'Rehabilitation Nursing'/exp

#91 #39 or #49

#92 #52 or #55 or #60 or #64 or #70 or #89

#93 #25 and #91 and #92

**Database: Cochrane**

#1 [Mh"Head and Neck Neoplasms"]

#2 "Neoplasms, Head and Neck" :ti,ab

#3 "Head, Neck Neoplasms" :ti,ab

#4 "Cancer of Head and Neck" :ti,ab

#5 "Head and Neck Cancer" :ti,ab

#6 "Cancer of the Head and Neck" :ti,ab

#7 "Upper Aerodigestive Tract Neoplasms" :ti,ab

#8 "UADT Neoplasms" :ti,ab

#9 "Neoplasm, UADT" :ti,ab

#10 "Neoplasms, UADT" :ti,ab

#11 "UADT Neoplasm" :ti,ab

#12 "Neoplasms, Upper Aerodigestive Tract" :ti,ab

#13 "Head Neoplasms" :ti,ab

#14 "Neoplasms, Head" :ti,ab

#15 "Neck Neoplasms" :ti,ab

#16 "Neoplasms, Neck" :ti,ab

#17 "Cancer of Head" :ti,ab

#18 "Head Cancer" :ti,ab

#19 "Cancer of the Head" :ti,ab

#20 "Cancer of Neck" :ti,ab

#21 "Neck Cancer" :ti,ab

#22 "Cancer of the Neck" :ti,ab

#23 or #1-#22

#24 [Mh"Otorhinolaryngologic Neoplasms"]

#25 "Neoplasm, Otorhinolaryngologic":ti,ab

#26 "Otorhinolaryngologic Neoplasm":ti,ab

#27 "Ootorhinolaryngeal Neoplasms":ti,ab

#28 "Neoplasm, Ootorhinolaryngeal":ti,ab

#29 "Neoplasms, Ootorhinolaryngeal":ti,ab

#30 "Ootorhinolaryngeal Neoplasm":ti,ab

#31 "Neoplasms, Otorhinolaryngological":ti,ab

#32 "Neoplasm, Otorhinolaryngological":ti,ab

#33 "Otorhinolaryngological Neoplasm":ti,ab

#34 "Otorhinolaryngological Neoplasms":ti,ab

#35 "Neoplasms, Otorhinolaryngologic":ti,ab

#36 "Otorhinolaryngeal Cancer":ti,ab

#37 "Cancer, Otorhinolaryngeal":ti,ab

#38 "Cancers, Otorhinolaryngeal":ti,ab

#39 "Otorhinolaryngeal Cancers":ti,ab

#40 or #24-#39

#41 HNC or HNSCC or SCCHN or OPSCC

#42 #23 or #40 or #41

#43 [Mh"Deglutition Disorders"]

#44 "Deglutition Disorder" :ti,ab

#45 "Disorders, Deglutition" :ti,ab

#46 "Swallowing Disorders" :ti,ab

#47 "Swallowing Disorder" :ti,ab

#48 "Dysphagia" :ti,ab

#49 "Oropharyngeal Dysphagia" :ti,ab

#50 "Dysphagia, Oropharyngeal" :ti,ab

#51 "Esophageal Dysphagia" :ti,ab

#52 "Dysphagia, Esophageal" :ti,ab

#53 or #43-#52

#54 [Mh"Motor activity"]

#55 "Strength training" :ti,ab

#56 or #54-#55

#57 [Mh"Exercise"]

#58 "Exercise training" :ti,ab

#59 or #57-#58

#60 [Mh"Exercise therapy"]

#61 "Exercise therapy" :ti,ab

#62 "Exercise program" :ti,ab

#63 "Physical exercise" :ti,ab

#64 or #60-#63

#65 [Mh"Occupational therapy"]

#66 "Exercise intervention" :ti,ab

#67 "Physical activity" :ti,ab

#68 or #65-#67

#69 "Pre-treatment swallowing intervention":ti,ab

#70 "Prophylactic swallowing therapy":ti,ab

#71 "Prophylactic swallowing rehabilication" :ti,ab

#72 "Prophylactic swallowing exercises":ti,ab

#73 "Pre-treatment care":ti,ab

#74 or #69-#73

#75 [Mh"Rehabilitation"]

#76 "Habilitation" :ti,ab

#77 #75 or #76

#78 [Mh"Rehabilitation Nursing"]

#79 "Nursing, Rehabilitation":ti,ab

#80 "Nursings, Rehabilitation":ti,ab

#81 "Rehabilitation Nursings":ti,ab

#82 #78 or #79 or #80 or #81

#83 #56 or #59 or #64 or #68 or #74 or #77 or #82

#84 #53 and #83

#85 #41 and #84

**Database: Ovid MEDLINE**

#1 'head and neck tumor'/exp

#2 'ear nose throat tumor':ab,ti

#3 'ear nose throat tumour':ab,ti

#4 'ENT tumor':ab,ti

#5 'ENT tumour':ab,ti

#6 'head and neck neoplasms':ab,ti

#7 'head and neck tumour':ab,ti

#8 'head neck tumor':ab,ti

#9 'head neck tumour':ab,ti

#10 'ORL tumor':ab,ti

#11 'ORL tumour':ab,ti

#12 'otorhinolaryngeal tumor':ab,ti

#13 'otorhinolaryngeal tumour':ab,ti

#14 'otorhinolaryngologic neoplasms':ab,ti

#15 'otorhinolaryngologic tumor':ab,ti

#16 'otorhinolaryngologic tumour':ab,ti

#17 'otorhinolaryngological tumor':ab,ti

#18 'otorhinolaryngological tumour':ab,ti

#19 'tumor, head and neck':ab,ti

#20 'tumour, head and neck':ab,ti

#21 HNC:ab,ti

#22 HNSCC:ab,ti

#23 SCCHN:ab,ti

#24 OPSCC:ab,ti

#25 or #1-#24

#26 'dysphagia'/exp

#27 'Aphagopraxia':ab,ti

#28 'deglutition difficulty':ab,ti

#29 'deglutition disorder':ab,ti

#30 'deglutition disorders':ab,ti

#31 'difficult deglutition':ab,ti

#32 'difficulty in swallowing':ab,ti

#33 'difficulty swallowing':ab,ti

#34 'dysphagias':ab,ti

#35 'swallowing difficult':ab,ti

#36 'swallowing difficultness':ab,ti

#37 'swallowing difficulty':ab,ti

#38 'swallowing disorder':ab,ti

#39 or #26-#38

#40 'swallowing'/exp

#41 'deglutition':ab,ti

#42 'oropharyngeal swallow':ab,ti

#43 'reflex, deglutition':ab,ti

#44 'swallow (deglutition) ':ab,ti

#45 'swallow (ingestion) ':ab,ti

#46 'swallow function':ab,ti

#47 'swallow reflex':ab,ti

#48 'swallowing reflex':ab,ti

#49 or #40-#48

#50 'Motor activity'/exp

#51 'Strength training':ab,ti

#52 or #50-#51

#53 'Exercise"/exp

#54 'Exercise training':ab,ti

#55 or #53-#54

#56 'Kinesiotherapy'/exp

#57 'Exercise therapy':ab,ti

#58 'Exercise program':ab,ti

#59 'Physical exercise':ab,ti

#60 or #56-#59

#61 'Occupational therapy'/exp

#62 'Exercise intervention':ab,ti

#63 'Physical activity':ab,ti

#64 or #61-#63

#65 'Pre-treatment swallowing intervention':ab,ti

#66 'Prophylactic swallowing therapy':ab,ti

#67 'Prophylactic swallowing rehabilication':ab,ti

#68 'Prophylactic swallowing exercises':ab,ti

#69 'Pre-treatment care':ab,ti

#70 or #65-#69

#71 'rehabilitation'/exp

#72 'functional readaptation':ab,ti

#73 'medical rehabilitation':ab,ti

#74 'readaption':ab,ti

#75 'readjustment':ab,ti

#76 'rehabilitation concept':ab,ti

#77 'rehabilitation engineering':ab,ti

#78 'rehabilitation potential':ab,ti

#79 'rehabilitation process':ab,ti

#80 'rehabilitation program':ab,ti

#81 'rehabilitation programme':ab,ti

#82 'rehabilitation, medical':ab,ti

#83 'rehabilitative treatment':ab,ti

#84 'resocialization':ab,ti

#85 'resocialisation therapy':ab,ti

#86 'resocialization':ab,ti

#87 'resocialization therapy':ab,ti

#88 'revalidation':ab,ti

#89 or #71-#88

#90 'rehabilitation Nursing'/exp

#91 #39 or #49

#92 #52 or #55 or #60 or #64 or #70 or #89

#93 #25 and #91 and #92

**Methodological quality**

**Table S1.**  **Methodological quality of the 7 included Randomized Controlled Trials**

| **First author, year** | **Q1** | **Q2** | **Q3** | **Q4** | **Q5** | **Q6** | **Q7** | **Q8** | **Q9** | **Q10** | **Q11** | **Q12** | **Q13** | **Total** |
| --- | --- | --- | --- | --- | --- | --- | --- | --- | --- | --- | --- | --- | --- | --- |
| **S. F. Hajdú (2021)** | **+** | **+** | **+** | **‐** | **‐** | **+** | **+** | **+** | **+** | **+** | **+** | **+** | **+** | **11/13** |
| **Jansen (2020)** | **+** | **+** | **+** | **-** | **-** | **-** | **+** | **+** | **+** | **+** | **+** | **+** | **+** | **10/13** |
| **Jansen (2021)** | **+** | **+** | **+** | **‐** | **‐** | **-** | **+** | **+** | **+** | **+** | **+** | **+** | **+** | **10/13** |
| **Wall (2020)** | **+** | **+** | **+** | **‐** | **‐** | **-** | **+** | **+** | **+** | **+** | **+** | **+** | **+** | **10/13** |
| **Baudelet (2023)** | **+** | **+** | **+** | **-** | **-** | **-** | **+** | **+** | **+** | **+** | **+** | **+** | **+** | **10/13** |
| **Pang (2023)** | **+** | **+** | **+** | **-** | **-** | **+** | **+** | **+** | **+** | **+** | **+** | **+** | **+** | **11/13** |
| **Starmer (2023)** | **+** | **+** | **+** | **-** | **-** | **-** | **+** | **+** | **+** | **+** | **+** | **+** | **+** | **10/13** |

**Abbr.: +, Positive quality assessment; -, Negative quality assessment;**

**?, Not clear; X, not applicable.**

1. Was true randomization used for assignment of participants to treatment groups?

2. Was allocation to treatment groups concealed?

3. Were treatment groups similar at the baseline?

4. Were participants blind to treatment assignment?

5. Were those delivering treatment blind to treatment assignment?

6. Were outcomes assessors blind to treatment assignment?

7. Were treatment groups treated identically other than the intervention of interest?

8. Was follow up complete and if not, were differences between groups in terms of their
follow up adequately described and analyzed?

9. Were participants analyzed in the groups to which they were randomized?

10. Were outcomes measured in the same way for treatment groups?

11. Were outcomes measured in a reliable way?

12. Was appropriate statistical analysis used?

13. Was the trial design appropriate. and any deviations from the standard Randomized Controlled Trials design (individual randomization, parallel groups) accounted for in the conduct and analysis of the trial?

**Table S2.** **Methodological quality of the 5 included Quasi-experimental studies.**

| **First author,  year** | **Q1** | **Q2** | **Q3** | **Q4** | **Q5** | **Q6** | **Q7** | **Q8** | **Q9** | **Total** |
| --- | --- | --- | --- | --- | --- | --- | --- | --- | --- | --- |
| **Cnossen (2014)** | **+** | **-** | **X** | **X** | **+** | **+** | **X** | **+** | **+** | **5/9** |
| **Shinn (2019)** | **+** | **-** | **X** | **X** | **+** | **+** | **X** | **+** | **+** | **5/9** |
| **Starmer (2018)** | **+** | **‐** | **X** | **X** | **+** | **+** | **X** | **+** | **+** | **5/9** |
| **Collins (2017)** | **+** | **+** | **+** | **+** | **+** | **+** | **+** | **+** | **+** | **9/9** |
| **Constantinescu**  **(2021)** | **+** | **‐** | **X** | **X** | **+** | **+** | **X** | **+** | **+** | **5/9** |

**Abbr.: +, Positive quality assessment; -, Negative quality assessment;**

**?, Not clear; X, not applicable.**

1. Is it clear in the study what is the 'cause' and what is the 'effect' (i.e. there is no confusion about which variable comes first)?

2. Were the participants included in any comparisons similar?

3. Were the participants included in any comparisons receiving similar treatment/care, other than the exposure or intervention of interest?

4. Was there a control group?

5. Were there multiple measurements of the outcome both pre and post the intervention/exposure?

6. Was follow up complete and if not, were differences between groups in terms of their follow up adequately described and analyzed?

7. Were the outcomes of participants included in any comparisons measured in the same way?

8. Were outcomes measured in a reliable way?

9. Was appropriate statistical analysis used?

**Table S3.** **Methodological quality of the 1 included Cohort studies.**

| **First author, year** | **Q1** | **Q2** | **Q3** | **Q4** | **Q5** | **Q6** | **Q7** | **Q8** | **Q9** | **Q10** | **Q11** | **Total** |
| --- | --- | --- | --- | --- | --- | --- | --- | --- | --- | --- | --- | --- |
| **Cnossen (2017)** | **+** | **X** | **+** | **+** | **+** | **+** | **+** | **+** | **+** | **-** | **+** | **9/11** |

**Abbr.: +, Positive quality assessment; -, Negative quality assessment;**

**?, Not clear; X, not applicable.**

1. Were subjects in each group have similar characteristics and come from the same overall study?

2. Were exposure factors measured in the same way that subjects were assigned to exposed and non-exposed groups?

3. Was the exposure factor assessment method effective and credible?

4. Are confounding factors considered?

5. Were the measures for controlling confounding factors in place?

6. Is it described that the subjects did not observe the results at the time of exposure or the beginning of the study?

7. Was the method for measuring the achievement indicators effective and reliable?

8. Were follow-up times reported and long enough to observe outcomes?

9. Was the follow-upomplete and if not, are the reasons for the loss of follow - up described and analyzed?

10. Were measures taken to address the issue of loss of follow-up?

11. Was the information analysis method appropriate?
