# Supplementary material for: Effectiveness of Home-Based Telerehabilitation Interventions for Dysphagia in Patients With Head and Neck Cancer: Systematic Review
Source: J Med Internet Res. 2023 Sep 8;25:e47324. doi: 10.2196/47324 (PMC10517384; doi:10.2196/47324)
Supplement: Multimedia Appendix 2 [file jmir_v25i1e47324_app2.docx]

**Table S1. Characteristics of the included studies**

| **Author, year**  **(country)** | **Study design** | **Study aims** | **Patients** | | **Treatment type** | **Intervention type** | **Outcome measurement** | **Outcomes** |
| --- | --- | --- | --- | --- | --- | --- | --- | --- |
|  |  |  | **Age (median)**  **Sex, n** | **Cancer Stage, n**  **Primary Cancer Site, n** |  |  |  |  |
| Cnossen et al.  2014,2017  (Netherlands) | Prospective clinical cohort study | 1. To explore the adherence to a 12-week home-based swallowing exercise program and swallowing exercise performance (by exercise levels and exercise categories) of HNC patients using Head Matters, a guided self-help exercise program. 2. To explore the predictors of swallowing exercise performance and barriers and facilitators of exercise adherence. 3. To investigate whether exercise performance was associated with HNC-specific QoL throughout the 12-week exercise program. | 1. Age: 61 2. Sex:   Male 39  Female 11 | 1. Stage I: 4   Stage II: 3  Stage III: 17  Stage IV: 26   1. Oropharynx: 30   Larynx: 15  Hypopharynx:  5 | (Chemo)  Radiotherapy Surgery | **Asynchronous**  **And**  **Synchronous Tele-rehabilitation**  Head Matters   1. **instruction leaflet**   **(b) instruction booklet (with DVD)**  **(c) website**  **(d)Telephone support and supervision** | Adherence  Swallowing exercise performance levels  Barriers and  facilitators of  exercise  adherence  QoL  **(EORTC-QLQ-H&N35)** | Identified barriers (decline in physical condition, treatment-related barriers) and facilitators (increase in physical condition, positive feelings).  Adherence to the home-based preventative exercise program was high (70%) but decreased subsequently (38%).  Combination with chemotherapy negatively affected exercise performance levels. |
| Baudelet et al. 2023  (Belgian) | RCT | To investigate the effect of three preventive swallowing exercise service delivery modes on patients' actual adherence.  (1) Diary-supported  (2) App-supported  (3) Therapist-support | 1. Age: 63 2. Sex:   Male  Paper group:14  App group:11  Therapist group:  10  Female  Paper group:35  App group:38  Therapist group:  40 | 1. Stage I-II:   Paper group:25  App group:22  Therapist group:28  Stage III-IV:  Paper group:24  App group:27  Therapist group:22   1. NA | (Chemo)  Radiotherapy | **Asynchronous**  **And/or Synchronous Tele-rehabilitation**   1. **Diary-supported PSE (paper group)** 2. **App-supported PSE (app group)** 3. **Therapist-supported PSE (therapist group)** | Visual analog scale **(VAS)**  Adherence | This RCT found a significant impact of service-delivery mode (therapist, paper, app), time (1–4 weeks) and the interaction of both on adherence. |
| Pang et al.  2023  (China) | RCT | The purposes of this study were to evaluate the effects of tele-rehabilitation training on swallowing and articulation function in patients with oral and oropharyngeal tumors after surgery and to compare the effect of tele-rehabilitation intervention on functional recovery. | a. Age:  IG: 59 CG: 60  b. Sex:  Male  IG: 17 CG: 14  Female  IG: 16 CG: 17 | a. NA  b. Anterior 2/3 of  tongue and floor of mouth:  IG: 11 CG: 11  Base of tongue:  IG: 4 CG: 3  Velopharyngeal  region:  IG: 5 CG: 4  Maxilla:  IG: 3 CG: 3  Mandible:  IG: 6 CG: 7  Buccal region:  IG: 5 CG: 4 | Surgery | **Asynchronous**  **And Synchronous**  **Tele-rehabilitation**  WeChat software  telerehabilitation collaboration | Maximum mouth opening  Swallowing ability  **(WST+MDADI)** | After 3 months of training, tele-rehabilitation training can improve the chewing and swallowing function of patients with oral and maxillofacial tumors.  The effect of tele-rehabilitation training on oral cancer patients is better than that of counterpart oropharyngeal cancer patients. |
| Hajdú et al.,  2021  (Denmark) | RCT | To investigate the effects of dual-mode swallowing exercise and progressive resistance exercise on swallowing safety, tube-feeding dependence, functional swallowing, mouth opening, physiological function, QoL, depression and anxiety of HNC patients. | 1. Age:   IG: 63 CG: 63   1. Sex:   Male  IG: 94 CG: 97  Female  IG: 26 CG: 18 | 1. Stage I:   IG: 27 CG: 28  Stage II:  IG: 25 CG: 17  Stage III:  IG: 19 CG: 24  Stage IVa:  IG: 42 CG: 43  Stage IVb:  IG: 7 CG: 3   1. Oropharynx:   IG: 68 CG: 72  Larynx:  IG: 26 CG: 28  Oral cavity:  IG: 10 CG: 5  Hypopharynx:  IG: 11 CG: 9  Unknown primary:  IG: 5 CG: 1 | (Chemo)  Radiotherapy | **Asynchronous**  **and Synchronous Tele-rehabilitation**   1. **Swallowing training sessions** 2. **Telephone** support and supervision   **(c) Booklet**  written  instructions | Aspiration, penetration  and residue  **(FEES+PAS+YPRS)**  Oral intake **(FOIS)**  Tube dependence  Mouth opening **(MID)**  Weight  QoL  **(EORTCQLQ-C30 +EORTCQLQ-H&N35+ MDADI)**  Pain **(NRS)**  Mood  **(MDI+ SCL-92 Anxiety subscale)**  Adherence | Prophylactic swallowing training during radiotherapy did not affect swallowing safety in patients with HNC based on penetration and aspiration measurements.  Possible explanations were as follows: short duration of intervention, lack of integration of exercise into survival, and small real differences between groups. |
| Jansen et al.,  2020, 2021  (Netherlands) | RCT | To investigate the effectiveness of a guided self-help exercise program (the "In Tune without Cords" application) on swallowing, speech and neck and shoulder problems in patients receiving treatment, and to investigate its cost-effectiveness. | 1. Age:   IG: 65 CG: 64   1. Sex:   Male  IG: 36 CG: 41  Female  IG: 10 CG: 5 | NA | (Chemo)  Radiotherapy  Surgery | **Asynchronous**  **and Synchronous Tele-rehabilitation**   1. **In Tune**   **without cords**  **Application**  **(b) DVDS, Booklet, images and videos**  **(c）Telephone support and supervision** | Swallowing problems  **(SWAL-QOL)**  Speech problems **(SHI)**  Shoulder problems  **(SDQ)**  QoL  **(EORTCQLQ-C30 +EORTCQLQ-H&N35+ EQ-5D-3 L+** **the EORTC QLU-C10D)**  Self-management  **(PAM)**  Costs  **(iMCQ + iPCQ)**  Adherence | The guided self-help exercise program improved swallowing and speech. Patients benefited most from the intervention within 6 months of surgery. |
| Shinn et al.,  2019  (USA) | Interim  study  analysis | 1. To describe the adaptability of the *PREPARE* intervention to mobile platform to enhance its potential use in communities of HNC patients.   b. To explore the influence of the website on patients' adherence to swallowing practice. | 1. Age: 63 2. Sex: NA | 1. Stages I & II: 14   Stages III & IV:  105   1. Larynx: 30   Oropharynx: 49  Hypopharynx: 3  Nasopharynx: 4  Oral cavity: 35  Other: 38 | (Chemo)  Radiotherapy | **Asynchronous**  **And**  **Synchronous**  **Tele-rehabilitation**   1. **The web based platform** 2. **Telephone** support and supervision | QoL **(MDADI)**  Adherence  Pain **(BPI)**  Patient engagement metrics | The web-based adherence  self-management program was significantly associated with increased adherence to swallowing exercises.  However, platform improvement in terms of navigability and usability was needed. |
| Wall et al.,  2020  (Australia) | RCT | To study the effect of service delivery mode on the clinical outcomes of HNC patients who completed preventive swallowing treatment. | 1. Age:   Total: 58  Clinician-directed:  59  Swallow IT: 60  Patient-directed: 60   1. Sex:   Male  Clinician-directed: 24  Swallow IT: 24  Patient-directed: 22  Female  Clinician-directed: 2  Swallow IT: 2  Patient-directed: 5 | NA | (Chemo)  Radiotherapy | **Asynchronous**  **And/or Synchronous Tele-rehabilitation**  **(a) Clinician directed face-to-face therapy**  **(b) SwallowIT application** | Aspiration, penetration and residue **(MBS/VFSS**+ **PAS)**  Oral intake **(FOIS)**  Maximum mouth opening **(MIMO)**  QoL **(MDADI** + **AQoL-6D** + **FACT-H&N)**  Nutritional status  **(PG-SGA)**  Mental health **(HADS)**  Fatigue **(MFI)**  Adherence  Patient satisfaction  **(FACIT-TSG)** | More patients preferred *SwallowIT* and the clinically guided model to the patient-guided model. *SwallowIT* might help provide future patients with intensive prophylactic swallowing therapy. |
| Starmer et al.,  2018  (USA) | Feasibility  study | To explore the feasibility of using a mobile health application to support patients and increase adherence to swallowing training after radiotherapy. | 1. Age: 62 2. Sex:   Male 28  Female 8 | 1. NA 2. Oropharynx: 30   Sinonasal: 2  Thyroid: 2  Larynx: 1  Oral cavity: 1 | (Chemo)  Radiotherapy Surgery | **Asynchronous**  **and Synchronous**  **Tele-rehabilitation**   1. **Vibrent™ application**   **(b) Face-to-face meeting** | Adherence  Fatigue  Pain  Weight | The Vibrent™ mobile application has potential to improve adherence to prophylactic swallowing training following radiotherapy for patients with HNC. |
| Starmer et al.  2023  (The USA) | RCT | Effect of mobile application on prophylactic swallowing exercise adherence and functional swallowing outcomes during HNC treatment. | 1. Age:   IG: 59 CG: 61   1. Sex:   Male  IG: 38 CG: 41  Female  IG: 6 CG: 6 | 1. Stage I~II:   IG: 32 CG: 35  Stage III~IV:  IG: 12 CG: 12   1. Oropharynx:   IG: 34 CG: 37  Other:  IG: 10 CG: 10 | (Chemo)  Radiotherapy | **Asynchronous**  **and Synchronous**  **Tele-rehabilitation**   1. **HNC Virtual Coach app**   **(b) Face-to-face meeting** | Adherence  Aspiration, penetration and residue **(MBS+PAS)**  Oral intake **(FOIS+PSS-HN)**  QoL **(MDADI+EORTC INFO-25)**  Weight | This study shows that a mobile health app can be used to enhance adherence with preventative swallowing exercises in HNC patients. In the subacute phase, the overall incidence of dysphagia was significantly reduced. |
| Collins et al.,  2017  (Australia) | Feasibility  study | 1. To compare the results of facility-based and home-based telemedicine service. 2. To explore the feasibility of software-based telemedicine service to provide home-based posttreatment intervention for HNC patients. 3. To compare the service and cost results and patient satisfaction. | 1. Age:   IG: 65 CG: 57   1. Sex:   Male  IG: 10 CG: 5  Female  IG: 5 CG: 5 | 1. Stage I:   IG: 2 CG: 2  Stage II:  IG: 6 CG: 5  Stage III:  IG: 2 CG: 5  Stage IV:  IG: 4 CG: 3   1. Oral:   IG: 6 CG: 6  Oropharyngeal:  IG: 5 CG: 5  Hypopharyngeal:  IG: 3 CG: 3  Other:  IG: 1 CG: 1 | (Chemo)  Radiotherapy Surgery | **Asynchronous and Synchronous Tele-rehabilitation**  **The software-based telehealth service**  **Video conferencing** | Patient satisfaction  **(FACIT-TSG)**  Cost analysis | Home telemedicine services were more efficient and less expensive for providing swallowing, speech, and nutrition interventions to patients after HNC treatment, thereby providing equitable access to high-quality health care for patients in the community. |
| Constantinescu et al., 2021  (Canada) | Quasiexperimental study | 1. To explore adherence to "Mobili-T" (a home-based remote swallowing exercise program) to treat HNC patients and to evaluate the potential factors related to adherence.   b. To explore differences in specific QoL results reported after swallowing treatment. | 1. Age: 62 2. Sex:   Male 15  Female 5 | 1. Stages I: 3   Stages II: 7  Stages III: 4  Stages IV: 3   1. Oropharynx : 16   Oral : 2  Unknown/other: 2 | (Chemo)  Radiotherapy Surgery | **Asynchronous**  **and Synchronous Tele-rehabilitation**  **(a) mHealth app**  **Swallowing**  **sEMG biofeedback**  **(b) Face-to-face meeting** | QoL  **(MDADI)**  Adherence Muscle activity  **(sEMG)** | Adherence to home-based swallowing treatment remained at ≥72% during treatment. In addition, 6 weeks of treatment improved the QoL of HNC patients with dysphagia. |

Abbreviations: AQoL-6D, the Assessment of Quality of Life-6D; BPI, Brief Pain Inventory; CNQ-SF, short-form Cancer Needs Questionnaire; CG, Control Group; DHI, Dysphagia Handicap Index; EORTC INFO-25, The European Organization for Research and Treatment of Cancer INFO-25; EORTCQLQ-C30, the European Organization for Research and Treatment of Cancer Quality of Life Questionnaire-Core 30; EORTCQLQ-H&N35, the European Organization for Research and Treatment of Cancer Quality of Life Questionnaire- Head and Neck 35; the EORTC QLU-C10D, the European Organization for Research and Treatment of Cancer QLU-C10D; EQ-5D-3 L, EuroQol five-dimensional questionnaire-3 L; FACIT-TSG, the Functional Assessment of Chronic Illness Therapy—Treatment Satisfaction—General; FACT-H&N, Functional Assessment of Cancer Therapy-Head and Neck; FEES, Fiberoptic Endoscopic Evaluation of Swallowing; FOIS, Functional Oral Intake Scale; HADS, the Hospital Anxiety and Depression Scale; HNC, head and neck cancer; IG, intervention group; iMCQ, Medical Consumption Questionnaire; iPCQ, Productivity Costs Questionnaire; MBS, modified barium swallow; MDADI, MD Anderson Dysphagia Inventory; MDI, Major Depression Index; MFI, Multidimensional Fatigue Inventory; MID, maximal interincisal distance; MIMO, maximum interincisor (mouth) opening; NA, not available; NRS, numerical rating scale; PAM, Patient Activation Measure; PAS, Penetration and Aspiration Scale; PSS-HN, Performance Status Scale—Head and Neck; QoL, quality of life; RCT, randomized controlled trial; SCL-92: Symptom Check List-92 Anxiety subscale; SDQ, Shoulder Disability Questionnaire; sEMG, system equipped with surface electromyography; SHI, Speech Handicap Index; SWAL-QoL, Swallow Quality of life; VAS, visual analog scale; VFSS, video fluorographic swallowing study; WST, Water Swallowing Test; YPRS, Yale Pharyngeal Residue Severity Rating Scale.

**Table S2. Characteristics of the interventions**

| **Author**  **(year)** | **Intervention description** | | **Sample size** | **Intervention timing** | **Intervention** **duration** | **Exercise frequency**  **Exercise intensity** |
| --- | --- | --- | --- | --- | --- | --- |
|  | **Intervention group** | **Control group** |  |  |  |  |
| Cnossen et al.,  2014, 2017  (Netherlands) | Pretreatment face-to-face consultation and 10-minute weekly telephone counseling sessions  **Head Matters** content**: (1) moving shoulders up and down, rotating shoulders back and forth, (2) swallowing hard and drinking water regularly, (3) humming with exaggerated sound volume and chin movement, sliding pitch up as high as possible (falsetto practice), and (4) pronouncing each syllable carefully**  Two formats: (a) web-based online exercises and (b) sample photographs and videos through a 15-minute instructional DVD. | NA | 94 (83) | During treatment and post treatment | 12 weeks | Exercise at least once a day  Low intensity: up to four types of exercise once a day.  Moderate intensity: four types of exercise, once or twice a day.  High intensity: four types of exercise at least twice a day,  15 min each time |
| Baudelet et al. 2023  (Belgian) | **Preventive swallowing exercises:**   1. **Tongue strengthening exercises (TSE) using the Iowa Oral Performance Instrument Patients** 2. **Chin-tuck against resistance (CTAR) exercises using the Swallowing Exercise Aid.** 3. **Diary-supported PSE (paper group)** 4. **App-supported PSE (app group)** 5. **Therapist-supported PSE (therapist group)** | NA | 148 (118) | Before  treatment and  during treatment | 4 weeks | 1. TSE:120 tongue presses per session,   divided into 12 sets of 10 repetitions. Pause for 30 s between every set.   1. CTAR: one session consisted of 30 sets of 5 repetitions for a total of 150 chin-tucks per day. |
| Pang et al.  2023  (China) | Tele-rehabilitation Collaboration Group  Communicate in a timely manner by sending text, pictures and videos  1. Sit up straight in front of a table to make sure your jaw is in the right position and can close smoothly.  2. Massage the surgical site and the upper and lower gums  3. Maximum mouth opening  4. Close your lips together (use your hands to help) and make cheek puffs  5. Try stretching and shrinking your tongue  6. Cut food into small pieces (cookies, diced cucumbers, diced carrots) and chew on the healthy side for 10 minutes | Standard of rehabilitation  exercises | 75 (64) | Post treatment | 6 months | Three times a day  Approximately 5 days a week |
| Hajdú et al.  2021 | 3 supervised meetings per week and weekly telephone calls to provide encouragement and consultation  **Swallowing training:**  **Moving tongue (back and forth, to cheek, to mouth corners, resistance), gargling, yawning, opening mouth (moving jaw side-to-side and forward and back), Valsalva maneuvers, Shaker exercise, Mendelsohn maneuver, Masako maneuver, and effortful swallow**  PRT training: Leg press, knee extension, chest press and lateral pull-down, and abdominal contraction and back extension | **Nonactive control group:**  Standard of care  **Active control group:**  An individually  tailored exercise plan | 235 (176) | During treatment and post treatment | 5–6 weeks | Swallowing practice:  7 days per week with up to 10 repetitions per exercise, 3 times per day  PRT Training:  Twice a week, 5-8 times each time |
| Jansen et al.  2020,2021  (Netherlands) | Participants received face-to-face instruction prior to treatment and weekly telephone support.  **Training** included **flexibility exercises for the head, neck and shoulders; range of motion exercises for the tongue, lips and chin; and additional lymphedema exercises (in the case of facial lymphedema).** | Standard of  care  Self-care education program | 233 (92) | Post  treatment | 12 weeks | 3 times a day,  once for 10-15 minutes |
| Shinn et al.  2019  (The USA) | A web-based platform  **Swallowing exercise videos** with verbal instructions**:**  **Shaker exercise, Mendelsohn maneuver, jaw stretching, supraglottic swallow, Masako maneuver, effortful swallow, and preventive exercises for mouth-opening difficulties**  The intervention emphasized personal control, proposed multiple strategies for each radiotherapy side effect, and encouraged patients to try new strategies on subsequent webpages if other strategies failed. | NA | 160 | Before  treatment, during treatment and post  treatment | 10 weeks | 2 sets daily  Repeat each set 3–5 times |
| Wall et al.  2020  (Australia) | Pretreatment face-to-face SLP education session and weekly telephone instruction  **Training content:**  **Sustained falsetto, tongue press, effortful swallow, Therabite^TM^ jaw stretch, Therabite^TM^ jaw strengthening**  (1) Clinician-guidance group  Exercise daily according to the exercise plan.  (2) Remote exercise group  Included instructional videos, images, and text descriptions of each exercise. Patients recorded the number of motor repetitions completed and the degree of difficulty perceived during each exercise. | Patient Guidance  Group  (self-guided exercise according to the exercise plan) | 79 (69) | During treatment and post  treatment | 6 weeks | 5 days a week  8 cycles" a day  1 cycle = 10 repetitions per exercise |
| Starmer et al.  2018  (The USA) | **The Vibrent™ mobile application:**  **Training content: effortful swallowing, Masako maneuver, Mendelsohn maneuver, effortful pitch glides, jaw stretches, jaw range-of-motion exercises**  Five modules: exercise videos, written instructions, reminders, exercise records, and educational guidance | NA | 36 | During treatment | 7 weeks | Twice daily  Pain threshold  Once daily  Repeat 10 times in 3 sets  Pain threshold  Repeat 10 times in 2 sets |
| Starmer et al.  2023  (The USA) | **“HNC Virtual Coach” mobile application**  **exercise content：**  **Jaw stretches,**  **the Effortful Swallow, the Mendelsohn Maneuver, and the**  **Masako Maneuver** | Paper logging control group  (written handouts) | 98(66) | During treatment | 7 weeks | 3 sets of 10 repetitions  Twice daily |
| Collins et al.  2017  (Australia) | Preintervention educational meeting: Demonstration of technical setup, test equipment connections, and interpretation of meeting tasks, in addition to instructions, a flashlight, mandibular protraction meter, and the use of a spatula  **Content of telemedicine module:**  SLP conducted video calls for assessment and **rehabilitation of swallowing and speech functions, nutrition management and examination of posttreatment symptoms**. | Standard care model:  Patients attended these meetings at the hospital and were evaluated by phone when they were unable to hold face-to-face meetings. | 30 | Post  treatment | NA | Standard care model:  Average of 27.5 minutes per meeting  Telemedicine mode:  Average of 23 minutes per meeting |
| Constantinescu et al., 2021  (Canada) | **Mobili-T's program:**   1. **Application training was divided into nine groups: three groups of regular swallowing, three groups of hard swallowing, and three groups of Mendelssohn maneuver swallowing.** 2. The SEMG device was attached below the chin, and SEMG signals were transmitted via Bluetooth to the Mobili-T application. The mHealth system also includes a clinician portal that allows remote monitoring of the number of completed workouts (the system defines a single swallow in terms of SEMG signals). | NA | 20 | Post  treatment | 6 weeks | Eight groups per day  9 swallowing trainings per group |

Abbreviations: NA, not available; sEMG, system equipped with surface electromyography; SLP, speech-language pathologists.
